# Licochalcone A activation of glycolysis pathway has an anti-aging effect on human adipose stem cells

**DOI:** 10.18632/aging.203734

**Published:** 2021-12-03

**Authors:** Yating Wu, Hao Wang, Jianbo Zhu, Haitao Shen, Hailiang Liu

**Affiliations:** 1 Key Laboratory of Xinjiang Phytomedicine Resource and Utilization of Ministry of Education, College of Life Sciences, Shihezi University, Shihezi 832003, China; 2 Institute for Regenerative Medicine, Shanghai East Hospital, Tongji University School of Medicine, Shanghai 200123, China

**Keywords:** licochalcone A, adipose-derived stem cells, cell proliferation, glycolysis, aging

## Abstract

Licochalcone A (LA) is a chalcone flavonoid of *Glycyrrhiza inflata*, which has anti-cancer, antioxidant, anti-inflammatory, and neuroprotective effects. However, no anti-aging benefits of LA have been demonstrated *in vitro* or *in vivo*. In this study, we explored whether LA has an anti-aging effect in adipose-derived stem cells (ADSCs). We performed β-galactosidase staining and measured reactive oxygen species, relative telomere lengths, and *P16*^ink4a^ mRNA expression. Osteogenesis was assessed by Alizarin Red staining and adipogenesis by was assessed Oil Red O staining. Protein levels of related markers runt-related transcription factor 2 and lipoprotein lipase were also examined. RNA sequencing and measurement of glycolysis activities showed that LA significantly activated glycolysis in ADSCs. Together, our data strongly suggest that the LA have an anti-aging effect through activate the glycolysis pathway.

## INTRODUCTION

Organism aging is accompanied by cell senescence caused by telomere shortening [1], increased reactive oxygen species (ROS) [2], oncogene activation [3], impaired nervous system functions, and a decline in immunity [4, 5]. The occurrence of various diseases is closely related to cell senescence [6], such as osteoporosis [7] and Parkinson’s disease [5]. Lipid profiles are associated with aging. Indeed, increased uptake of specific lipids promotes longevity and ameliorates disease phenotypes *in vivo* [8]. Moreover, lipid profiles have been invaluable to identify the nine denominators of aging: telomere attrition, genome instability, epigenetic alterations, mitochondrial dysfunction, deregulated nutrient sensing, altered intercellular communication, loss of proteostasis, cellular senescence, and adult stem cell exhaustion [9]. Therefore, inhibiting replicative senescence of cells is an important consideration to improve health.

Stem cell exhaustion is a critical factor of aging. Adipose-derived stem cells (ADSCs), which are obtained from adipose tissue, exhibit a high proliferative ability, rapidly self-renew, and can be directed to differentiate into osteoblasts, fibroblasts, and nerve cells [10]. Extensive research has shown that ADSCs play major roles in aging, such as increasing the superoxide dismutase level and decreasing the malondialdehyde content of aging rats [11]. Additionally, ADSCs promote myelin sheath regeneration and reduce loss of nerve functions in mice [12]. Furthermore, injection of an Alzheimer’s disease mouse model with a certain dose of ADSCs promotes microglial cell activation and nerve regeneration by ADSCs homing to the lesion site and differentiating into nerve cells [13, 14]. However, the proliferative ability of ADSCs decreases in aged animals [15] and young ADSC transplants show significantly higher bone regeneration for osteoporosis treatment [16]. Regardless, ADSCs are a suitable cell source to research aging-related problems.

Natural small molecule compounds are widely used in clinical studies. Recently, increasing attention has been focused on the molecular mechanisms related to aging controlled by an epigenetic-modulating diet with polyphenols [17]. For example, resveratrol plays an important role in anti-aging by activation of deacetylases [18], whereas quercetin induces cytochrome c expression in the brain [19].

The potential clinical applications of numerous bioactive compounds in licorice were recently investigated. Licochalcone A (LA), a characteristic chalcone extracted from the root of *Glycyrrhiza inflata* [20], has bioactive functions and exerts anti-tumor [21], antioxidant [22], anti-obesity [23], and neuroprotective effects [24] and also shows anti-cancer effects by inhibiting glioma stem cell proliferation [25]. LA also exhibits anti-obesity effects [26], inhibits thrombus formation [27], and exerts antioxidant activities by regulating nuclear factor-erythroid 2-related factor 2 (Nrf2) [28]. Another study demonstrated that LA shows anti-inflammatory effects in IL-1β-stimulated chondrocytes [29]. Furthermore, treatment with LA promotes strong osteogenic differentiation and mineralized formation of cell aggregates [30]. Thus, LA is a component of licorice with various bioactivities. Although various pharmacological activities of LA have been reported, the value of LA as an adjuvant for anti-aging remains to be determined.

In this study, we investigated whether LA had an effect on the proliferative and differentiation abilities of hADSCs and examined the underlying anti-aging mechanisms. Additionally, we determined whether treatment with LA improved cell proliferation by RNA sequencing (RNA-seq) analysis and measurement of glycolysis activities.

## RESULTS

### LA ameliorates replicative senescence of hADSCs

Samples of hADSCs were obtained from subcutaneous adipose tissues and the cells had been identified by our previous work [31], a characterization of the multipotency showed that a state of aging after P9 as evidenced by a gradual increase in size and irregular shape at P9 and P16. Additionally, expression of cell senescence-related molecular marker *P16^ink4a^* was increased significantly, telomeres were shortened, and the number of SA-β-gal-positive cells was increased significantly at P16. Therefore, we chose aging state cells to research the anti-aging effect of LA [31].

To evaluate LA cytotoxicity, hADSC were treated with various concentrations of LA for 24 h and then evaluated for cell viability. The results indicated that LA (25 μmol) significantly increased hADSC viability compared with Ctrl treatment (p<0.05) (Supplementary Figure 1A).

Furthermore, growth curves of various passages displayed an initial lag phase of 2 days, followed by an exponential log phase from 3 to 5 days, and then cells entered a plateau phase at 6-7 days (Supplementary Figure 1B). The results showed that LA (25 μmol) promotes cell proliferation compared with Ctrl treatment on the fourth day (p<0.05).

**Figure 1:**
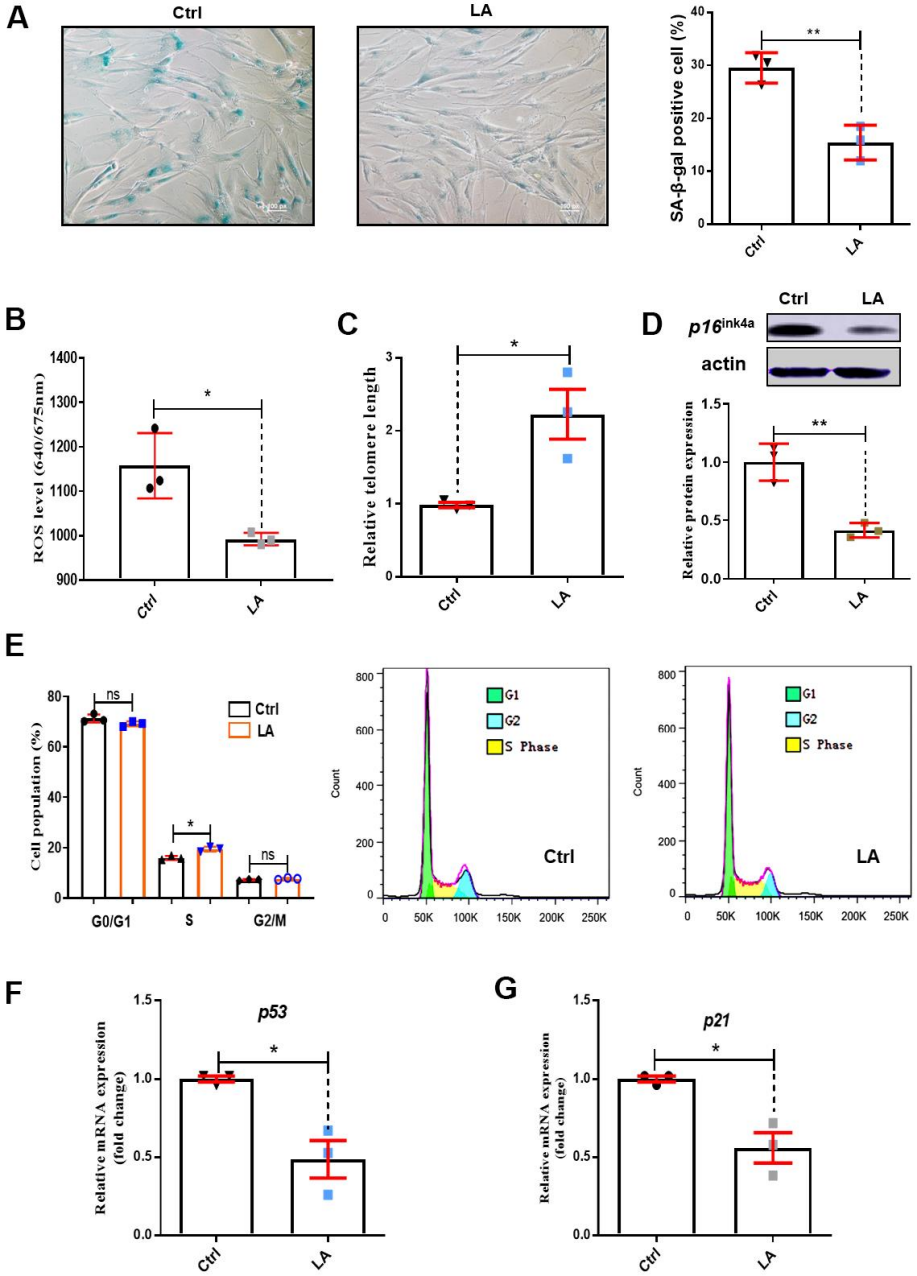
**Effects of LA on hADSC viability.** (**A**) SA-β-gal staining of hADSCs (Scale bar: 100 μm). The relative intensity of ROS was detected by a microplate reader. (**B**) Effects of LA on ROS. (**C**) Relative telomere lengths of hADSCs. (**D**) Relative protein expression of senescence gene *P16* compared with the control. (**E**) Effects of LA on cell cycle. (**F**, **G**) Relative mRNA expression of senescent genes *P21* and *P53* compared with control. Data are presented as the mean ± SD of three independent experiments. *p < 0.05, **p < 0.01 compared with untreated cells.

To examine the effect of LA on senescence, we performed SA-β-gal assays. SA-β-gal staining revealed significantly fewer senescent cells in the LA treatment group compared with the Ctrl group (p<0.01) (Figure 1A).

ROS levels are important to regulate cell proliferation activity. Increased ROS levels lead to cell senescence [32]. As shown in Figure 1B, treatment with LA (25 μmol) reduced ROS levels in hADSCs compared with the Ctrl (*P*<0.05). The length of telomeres inevitably shrinks during the process of aging by telomere regulation of cell replication [33] and cell senescence [34]. Telomere shortening was suppressed in LA-treated hADSCs compared with the Ctrl (*P*< 0.05) (Figure 1C). *P16*^ink4a^ is a marker of senescence [35] and senescence occurs by inactivation of suppressor elements, which enhances expression of p16 [36]. Western blotting showed that LA (25 μmol) treatment inhibited protein expression of *P16*^ink4a^ in aged hADSCs compared with the Ctrl (Figure 1D).

Cell cycle arrest results in genomic instability and premature aging [37], and delayed S phase progression contributes to genome instability [38]. Cell cycle analysis showed that LA (25 μmol) treatment caused a decrease in cells in G0/G1 phase and a significant increase in S phase cells (p<0.05) compared with Ctrl cells (Figure 1E).

In normal cells, the P53 tumor suppressor maintains mitochondrial respiration. Overexpression of *P53* drastically reduced mitochondrial Ca^2+^ transients in stimulated cells, causing mitochondrial dysfunction [39]. RT-qPCR showed that LA (25 μmol) treatment resulted in significantly reduced mRNA expression of *P53* (p<0.01) compared with Ctrl (Figure 1F). The senescence-associated secretory phenotype develops because of cellular senescence. P21 is a secretory factor that regulates senescence [40]. We found that LA (25 μmol) treatment also significantly reduced (p<0.05) the mRNA expression of *P21* compared with the Ctrl (Figure 1G).

### Effect of LA on the differentiation capabilities of hADSCs

To assess the anti-aging effect of LA (25 μmol), we examined cell differentiation abilities. Osteogenic and adipogenic differentiation indicates the differentiation potentials of stem cells [41] and osteogenesis of aged MSCs is compromised significantly [42]. Unbalanced adipogenic and osteogenic differentiation of human mesenchymal stem cells promotes senescence [43].

**Figure 2:**
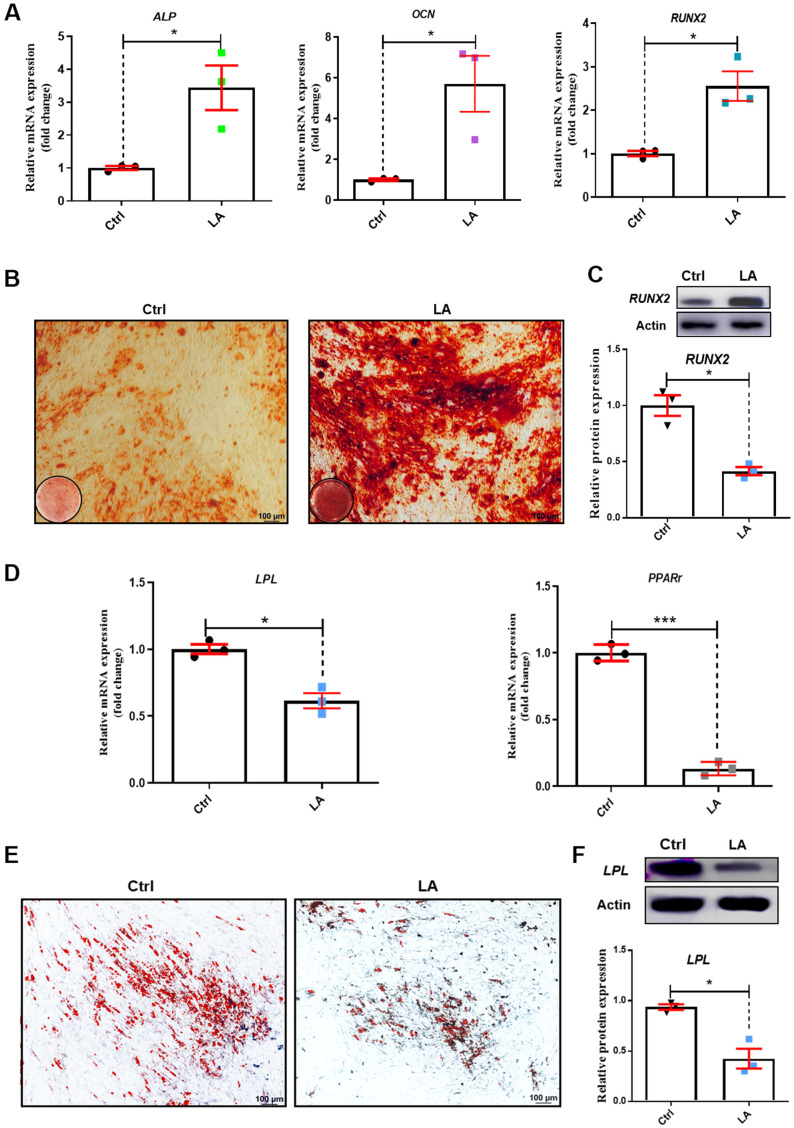
**Effects of LA on hADSC differentiation ability.** (**A**) qRT-PCR of mRNA levels of the osteogenic differentiation markers *ALP*, *OCN*, and *RUNX2*. (**B**) Alizarin Red staining of hADSCs. (**C**) Western blot in hADSCs treated as indicated. (**D**) qRT-PCR of mRNA levels of the adipogenic differentiation markers *PPARr* and *LPL*. (**E**) Oil Red O staining of hADSCs. (**F**) Western blot in hADSCs treated as indicated. Data are presented as the mean ± SD of three independent experiments. *p < 0.05, **p < 0.01, ***p < 0.001 compared with untreated cells.

We next examined osteogenic differentiation in hADSCs treated with LA (25 μmol) by evaluating mRNA levels of the marker genes alkaline phosphatase (*ALP*), alkaline osteocalcin (*OCN*), and runt-related transcription factor 2 (*RUNX2*) [44]. Compared with the Ctrl, LA (25 μmol) treatment increased the mRNA expressions of *ALP* (p<0.05), *OCN* (p<0.01), and *RUNX2* (p<0.01) (Figure 2A). Alizarin Red staining revealed that LA (25 μmol) promoted the formation of mineralized nodules compared with the Ctrl (Figure 2B). Western blot analysis showed that LA (25 μmol) upregulated the protein expression of RUNX2 (p<0.05) compared with the Ctrl (Figure 2C).

We also evaluated adipogenic differentiation by evaluating the mRNA levels of the marker genes peroxisome proliferator-activated receptors γ (*PPARγ*) and lipoprotein lipase (*LPL*) [45]. LA (25 μmol) treatment decreased the mRNA expression of *PPARγ* (p<0.01) and *LPL* (p<0.05) compared with the Ctrl (Figure 2D). Oil Red O staining showed that LA (25 μmol) treatment resulted in fewer fat droplets compared with the Ctrl (Figure 2E). Western blot analysis showed that LA (25 μmol) downregulated the protein expression of LPL (p<0.01) compared with the Ctrl (Figure 2F). These results demonstrated that LA (25 μmol) promotes hADSC differentiation toward osteogenesis and attenuates adipogenic differentiation.

### Effect of LA on glycolysis/gluconeogenesis pathways in hADSCs

**Figure 3:**
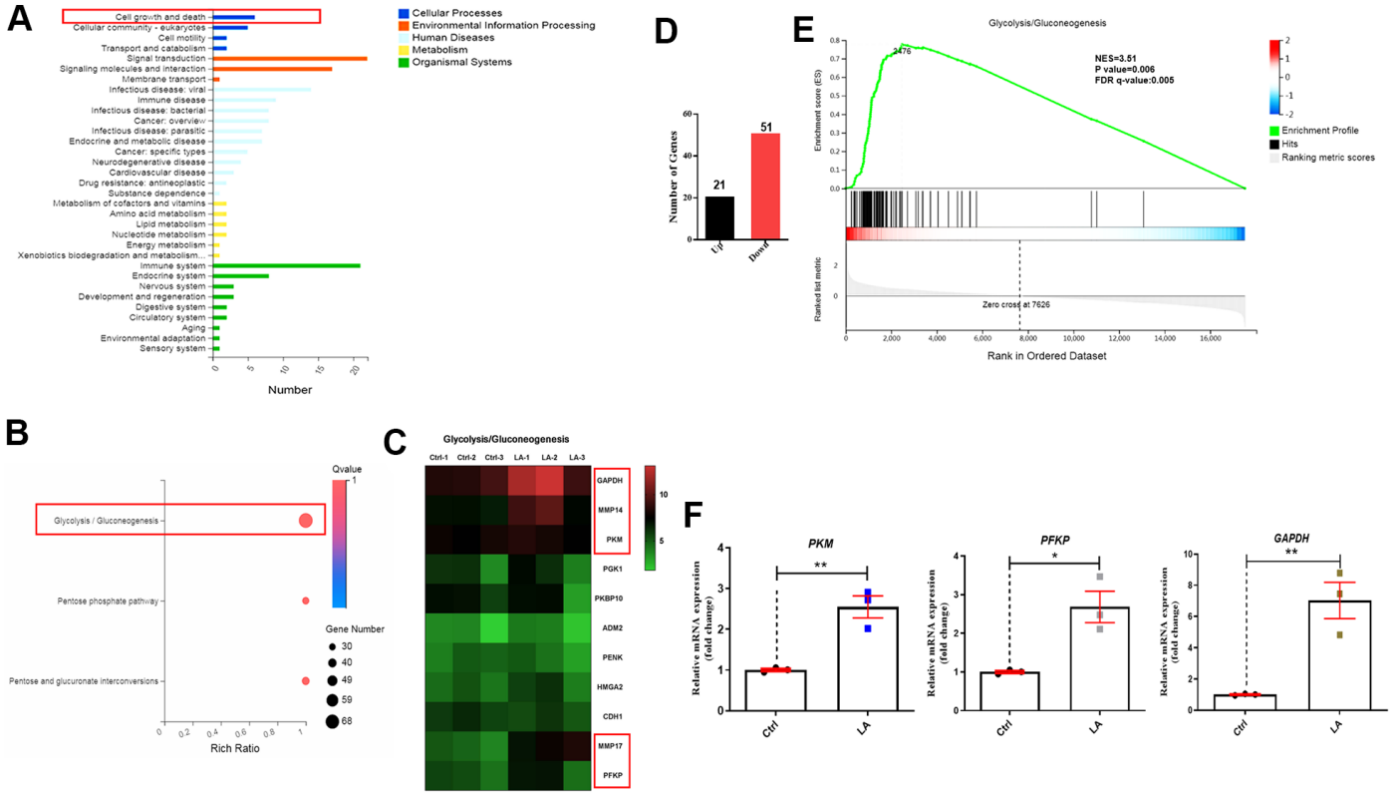
**Mechanism of LA in regulation of hADSC proliferation determined by RNA-seq.** (**A**) Classification map of KEGG pathway analysis. (**B**) Bubble diagram of glycolysis/gluconeogenesis signaling pathways regulated by LA. (**C**) Heatmap of differentially expressed genes related to glycolysis/gluconeogenesis signaling pathways. (**D**) The differentially expressed genes. (**E**) Enrichment plots of gene expression signatures for the glycolysis/gluconeogenesis signaling pathway. (**F**) qRT-PCR of the relative mRNA expression of *PKM*, *PFKP*, and *GAPDH* in LA-treated hADSCs. Data are presented as the mean ± SD of three independent experiments. Statistical significance was determined by the unpaired t-test. *p < 0.05, **p < 0.01 compared with the Ctrl.

On the basis of RNA-seq to analyze the specific mechanism, a classification map of KEGG pathway analysis revealed that 4 signaling pathways related to cell growth and death were regulated by LA (25 μmol) (Figure 3A). Furthermore, a bubble diagram showed that glycolysis/gluconeogenesis signaling pathways were regulated by LA (Figure 3B) and a heatmap showed differentially expressed genes related to glycolysis/gluconeogenesis signaling pathways (Figure 3C). Heatmap analysis showed that the expressions of *GAPDH*, *MMP14*, *PKM*, *MMP17*, and *PFKP* genes, which are related to the glycolysis/gluconeogenesis signaling pathway, were upregulated in the LA group compared with the Ctrl group. Examination of differentially expressed genes revealed 21 upregulated genes and 51 downregulated genes in the LA -treated group compared with the Ctrl group (Figure 3D). Furthermore, gene set enrichment analysis (GSEA) of the differentially expressed genes revealed that LA treatment activated the glycolysis/gluconeogenesis signaling pathways (Figure 3E).

To verify the RNA-seq results, we examined the mRNA levels of *PKM*, *PFKP*, and *GAPDH* in hADSCs treated with LA (25 μmol). Treatment with LA significantly increased the mRNA expressions of *PKM* (p<0.05), *PFKP* (p<0.05), and *GAPDH* (p<0.01) in hADSCs compared with the Ctrl group (Figure 3F). Western blot analysis also showed that LA upregulated the protein expression of *MMP*14 (p<0.01), *PFKP* (p<0.01) compared with the Ctrl and LA cannot regulate the protein expression of *PKM* (Supplementary Figure 2).

### LA promotes hADSC proliferation by activating the glycolysis pathway

To evaluate the effect of LA on glycolysis/gluconeogenesis pathways in hADSCs, we used extracellular flux analyzes to monitor the extracellular acidification rate (ECAR) and oxygen consumption rate (OCR) [46, 47], which indicated glycolysis pathway activity and mitochondrial respiration, respectively.

**Figure 4:**
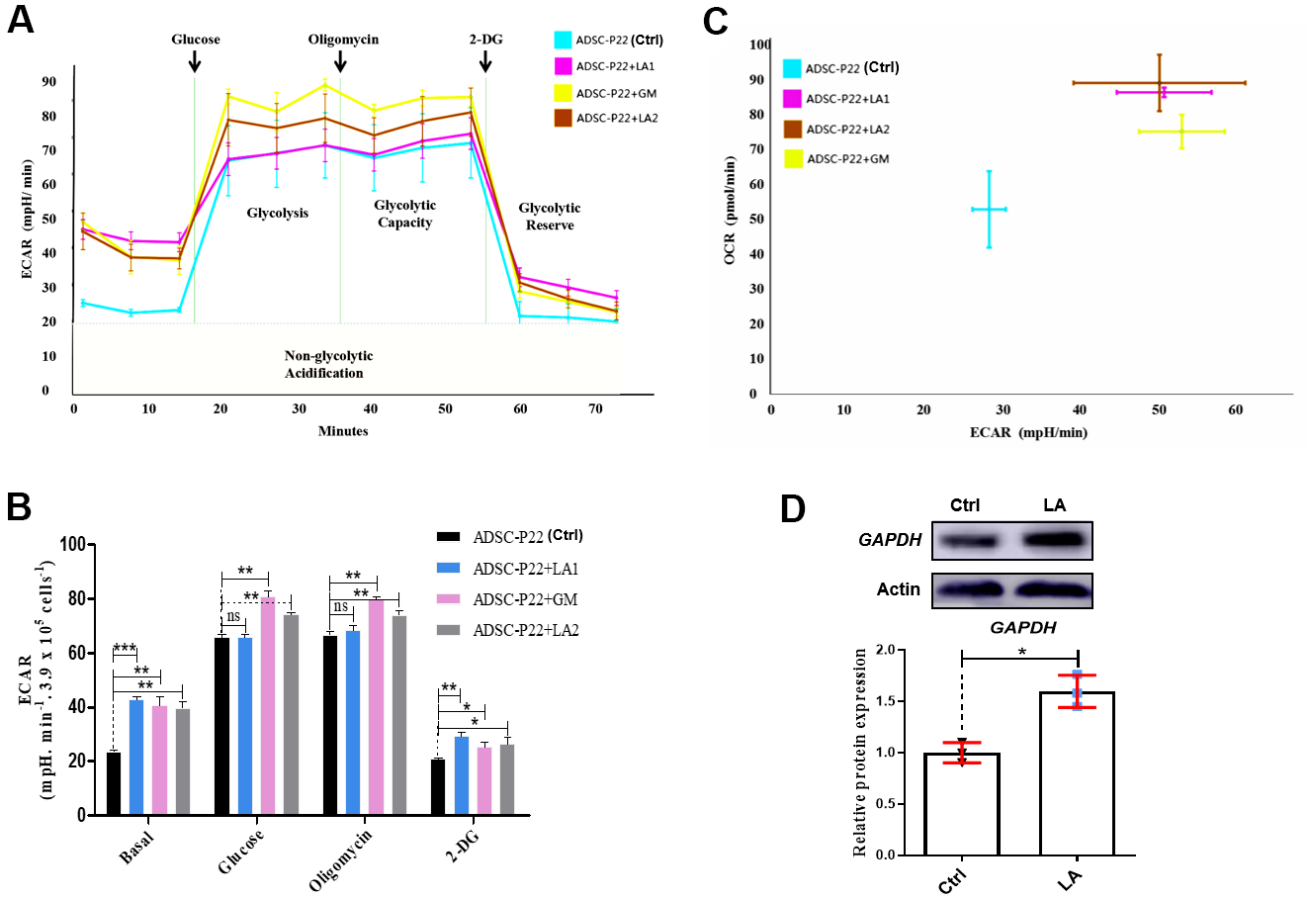
**Measurements of glycolysis activities in hADSCs.** (**A**) ECAR in hADSCs at each phase of the glycolysis pathway. (**B**) Statistical analysis of ECAR at each phase. (**C**) Relationship between ECAR and OCR in the glycolysis pathway of hADSCs. GM: Metformin. (**D**) Western blotting of GAPDH protein expression. Data are presented as the mean ± SD of three independent experiments. Statistical significance was determined by the unpaired t-test. *p < 0.05, **p < 0.01, ***p < 0.001 compared with the Ctrl.

Following LA treatment of hADSCs, glucose consumption was increased (Figure 4A). ECAR in hADSCs at each phase in the glycolysis pathway showed that basal, glucose, oligomycin, and 2-DG (2-Deoxy-D-glucose, one of the glycolysis inhibitors) phases were significantly higher in LA1 and LA2 groups than in the Ctrl (*P*<0.01) (Figure 4B). Additionally, when treated with LA (25 μmol), the relationship between ECAR and OCR showed a positive correlation and was higher than in the Ctrl (Figure 4C). Glycolysis levels showed that LA activated the glycolysis pathway by significantly improving ECAR and OCR in aged hADSCs compared with the Ctrl. Previous RNA-seq data suggested that LA can increases the expression of *GAPDH*, a critical regulation enzyme in the glycolysis pathway, and the upregulation of GAPDH expression can activate glycolysis progression [48]. Western blot analysis showed that GAPDH protein level was increased in hADSCs treated with LA (p<0.05) compared with the Ctrl (Figure 4D).

Other research found that AMPK (AMP-activated protein kinase) has been shown to regulate the senescence [49], which have reduced the expression of the epigenetic factor p-AMPK (phospho-AMP-activated protein kinase) in aged mice [50]. AMPK can mediate increase of glycolysis in human cells [51]. Western blot analysis also showed that LA (25 μmol) upregulated the protein expression of *p-AMPK* (p<0.01) compared with the Ctrl and cannot regulate the protein expression of *AMPK* (Supplementary Figure 3).

## Discussions

Young hADSCs are an ideal source of adult stem cells in clinical applications. However, the continuous culture of mesenchymal stem cells results in cell senescence. In this study, we examined the potential anti-aging effects of LA (25 μmol) on senescent hADSCs. Related evaluations confirm the regulation of LA on aging. The expression of *P16^INK4A^*, which regulates senescence, was downregulated by LA [52]. ROS levels are increased in aging cells, and increased ROS levels inhibit cell proliferation [53]. Our results showed that LA decreased the ROS level in hADSCs. Telomeres become shorter in senescent cells [54]. LA can protect telomeres from shortening in hADSCs during proliferation. Osteogenic and adipogenic differentiation is also a critical aging indicator [55]. During mesenchymal stem cell aging, bone mass decreases and bone marrow adiposity increases [56].

We performed osteogenic and adipogenic differentiation assays and found that LA (25 μmol) promoted osteogenesis differentiation and attenuated adipogenic differentiation of hADSCs. Although the four signaling pathways regulated by LA through RNA-seq analysis mainly involved cell growth and death but not osteogenic and adipogenic differentiation. This may be that the increase of osteogenic differentiation and the decrease of adipogenic differentiation in hADSCs caused by LA treatment is only a phenotype in cellular senescence.

Our RNA-seq results suggest that LA (25 μmol) activates the glycolysis signaling pathway by promoting *GAPDH*, *PKM*, *MMP14*, and *PFKP* gene expression. The glycolysis pathway is linked to cell growth and proliferation [57], so activation of the glycolysis pathway promotes cell viability and ameliorates aging [58]. *GAPDH*, *PKM*, *MMP14*, and *PFKP* are important factors in the glycolysis signaling pathway. For example, GAPDH activates the glycolysis pathway [59]. Increased expression of the glycolysis enzyme PKM facilitates a metabolic shift to glycolysis [60], and PFKP plays important roles in cellular glucose metabolism [61]. The results showed that LA activates the *p-AMPK*, which also involved in the senescence. Glycolysis inhibitor blocked LA-induced *AMPK* phosphorylation (Supplementary Figure 4A), and inhibition of Glycolysis blocked LA- attenuate fewer senescent cells (Supplementary Figure 4B) and expression of p16 gene from hADSCs (Supplementary Figure 4C).

In conclusion, our results suggest that the anti-aging effects of LA (25 μmol) are associated with induction of the glycolysis pathway. As is known to all, glycolysis is a critical pathway in glucose metabolism that provides intermediates for energy generation [62]. Such as upregulation of glycolysis is a major characteristic of provides energy to support rapid proliferation [63]. Glycolysis also regulates the activation of fibrogenesis in the aged lung *in vivo* and *in vitro* [57]. So, glycolysis is linked to cell growth and the findings showed the anti-aging property of LA by activating the glycolysis pathway. These findings may have broad implications for therapeutic that LA has an anti-aging effect on human adipose stem cells.

## MATERIALS AND METHODS

### Cell culture and viability assay

LA (C21H22O4; MW: 338; ≥98% pure, HPLC grade) was purchased from Chengdu Push Biotechnology Co. Ltd. (Sichuan, China) and stored at 2–8° C in a dark, dry place. The stock solution concentration was 100 mmol in dimethyl sulfoxide (DMSO). The final DMSO concentration did not exceed 0.1% in the culture medium. hADSCs at passage (P) 22 (late passage) were cultured in a humidified atmosphere with 5% CO_2_ at 37° C in DMEM/F12 (Thermo Fisher Scientific, Waltham, MA, USA) supplemented with 10% FBS and 100 U/mL penicillin and streptomycin.

Cell viability and growth kinetics were measured by CCK-8 assays. Briefly, cells were seeded in 96-well culture plates and treated for 24 h with various concentrations of LA (0, 25, 50, and 100 μmol for the cell viability assay; 25 μmol for the growth kinetics assay). Next, 10 μL CCK-8 solution was added to each well, followed by incubation at 37° C for 2 h. Cell viability was measured using a microplate reader by recording the absorbance of each well at 450 nm.

### SA-β-gal assay

hADSCs were cultured in 24-well plates overnight and then treated with 25 μmol LA or the vehicle control for 24 h. Next, the cells were fixed and treated with SA-β-gal kit solution. Cell staining was examined by fluorescence microscopy.

### ROS assay

hADSCs were treated with 25 μmol LA or the vehicle control for 24 h and then incubated with ROS reagent in the dark at room temperature for 30 minutes (min). ROS levels were determined using a multi-mode microplate reader.

### qRT-PCR analysis

Telomere lengths in hADSCs were measured by qRT-PCR. hADSCs were seeded in six-well plates and treated with 25 μmol LA and the vehicle control (Ctrl) for 24 h. Briefly, total mRNA was extracted using Trizol (Thermo Fisher Scientific) and then reverse transcribed into cDNA using a reverse transcription kit. Total DNA was extracted using a DNA extraction kit. The PCR protocol was as follows: predenaturation at 95° C for 3 min followed by 43 cycles of denaturation at 95° C for 10 s, annealing at 55° C for 30 s, and extension at 72° C for 30 s. *36B4* was used as a Ctrl. qRT-PCR was performed using the Taq-Man real-time PCR system (Thermo Fisher Scientific) and SYB-Green real-time PCR system (TaKaRa). Relative gene expression levels were normalized to *ACTB* and calculated by the 2^-ΔΔCT^ method. Primer sequences are listed in Table 1.

**Table 1:** The primer of qRT-PCR.

**Gene**	**F (5’-3’)**	**R (5’-3’)**
*h-P21*	TTAGCAGCGGAACAAGGA	AAGACAACTACTCCCAGCCC
*h-P53*	TGCATTTTCACCCCACCCTT	ACACAGGTGGCAGCAAAGTT
*h-ALP*	GATGGCCTGAACCTCATCGA	AGTTCGGTCCGGTTCCAGAT
*h-RUNX2*	TGGCCGGGAATGATGAGA	TGAAACTCTTGCCTCGTCCG
*h-OCN*	GGACTGTGACGAGTTGGCTGAC	TGCCTGGAGAGGAGCAGAACTG
*h-LPL*	TGTATGAGAGTTGGGTGCCAAA	GCCAGTCCACCACAATGACAT
*h-PPARr*	TGCAAGGGTTTCTTCCGGA	GCAAGGCATTTCTGAAACCG
*h-PFKP*	CGGAAGTTCCTGGAGCACCTCTC	AAGTACACCTTGGCCCCCACGTA
*h-GAPDH*	ATCAGCAATGCCTCCTGCAC	TGGTCATGAGTCCTTCCACG
*h-PKM*	ATGTCGAAGCCCCATAGTGAA	TGGGTGGTGAATCAAGTCCA
*h-actin*	ACCCACACTGTGCCCATCT	ATGTCACGCACGATTTCCC
*h-telomere*	GGTTTTTGAGGGTGAGGGTGAGGGTGAGGGTGAGGGT	TCCCGACTATCCCTATCCCTATCCCTATCCCTATCCCTA
*h-36B4*	CAGCAAGTGGGAAGGTGTAATCC	CCCATTCTATCATCAACGGGTACAA

### Osteogenic and adipogenic differentiations assays

Briefly, cells were cultured in DMEM/F12 containing 25 μmol LA or Ctrl. At 70% confluence, cells were treated with OriCell™ differentiation media with 25 μmol LA maintained throughout the differentiation period. Alizarin Red staining was performed on day 25 for osteogenesis and Oil Red O staining on day 14 for adipogenesis. Images were obtained by microscopy.

### RNA-seq analysis

RNA-seq was performed independently and uniformly on hADSCs treated with LA (25 μmol) or Ctrl in duplicate and cultured for 6 days in a humidified atmosphere with 5% CO_2_ at 37° C in DMEM/F12 supplemented with 10% FBS and 100 U/mL penicillin and streptomycin. Clean reads were aligned to the reference gene sequence using bowtie-2 and the gene expression levels of each sample were calculated. Differentially expressed gene (DEG) detection was conducted by the DEG seq method [62]. The statistical results were based on the ma-plot method. The number of reads of specific genes obtained from the sample was randomly sampled, and then *p*-values were calculated in accordance with the normal distribution and corrected to q-values. To improve the accuracy of DEGs, genes with a different multiple of more than double and a q-value of ≤0.001 were defined and screened as significant DEGs. RNA sequencing data generated from this study have been deposited in NCBI GEO (https://www.ncbi.nlm.nih.gov/geo) under the accession number GSE144105.

### Western blot analysis

Briefly, hADSC were treated with 25 μmol LA or Ctrl for 24 h and 48 h (or 80 h) then lysed using RIPA buffer containing protease and phosphatase inhibitors at 4° C, followed by centrifugation for 10 min. The supernatant was collected and sample buffer (5×) was added at a ratio of 5:1. Samples were mixed well and boiled for 10 min, followed by storage at −40° C. Proteins were separated by 10% SDS polyacrylamide gel electrophoresis and transferred to PVDF membranes that were blocked by incubation with 2.5% dry skim milk, followed by overnight incubation with primary antibodies diluted in 2.5% dry skim milk at 4° C.

The following antibodies were used: monoclonal rabbit anti-*RUNX2*, anti-*LPL*, anti-P16^ink4a^, anti-*GAPDH*, anti-*MMP14*, anti-*PKM*, anti-*PFKP*, anti-*AMPK*, anti-*p- AMPK* (Abcam) at 1:1000 dilutions. The blots were then incubated with the secondary mouse or rabbit antibodies at room temperature for 1 h. Proteins were detected using the BioSpectrum 600 system. The western blots repeated 3 independent experiments.

### Measurement of glycolysis activities

The oxygen consumption rate (OCR; pmoles/min) and extracellular acidification rate (ECAR; mpH/min) were measured using Seahorse XF96 extracellular flux analyzers (Seahorse Bioscience, Billerica, MA, USA) in accordance with the manufacturer’s instructions [63]. hADSCs were seeded in an XF96 cell culture microplate. Twenty-four hours (h) later, DMEM/F12 medium was changed to Agilent Seahorse XF base medium (Agilent Technologies) supplemented with GlutaMAX™ (Thermo Fisher Scientific). The cells were cultured for 1 h in a CO_2_-free 37° C incubator and then subjected to a Seahorse XF Cell Mito Stress Test or Seahorse XF Glycolysis Stress Test. LA concentrations were 6.25 μmol (LA1) and 25 μmol (LA2) for the glycolysis test. Measurements were recorded at the intervals indicated in the test protocols.

### Statistical analysis

Statistical analysis was performed using GraphPad Prism 5.0. Data are expressed as the mean ± standard deviation (SD). Statistical comparisons of two groups were made by the unpaired t-test. Statistical comparisons of more than two groups were made by analysis of variance (ANOVA). A two-tailed *P*-value of *P*<0.05 was considered statistically significant and *P*<0.01 was considered extremely statistically significant.

## Supplementary Material

Supplementary Figures
