# Supplementary material for: Licochalcone A activation of glycolysis pathway has an anti-aging effect on human adipose stem cells
Source: Aging (Albany NY). 2021 Dec 3;13(23):25180–94. doi: 10.18632/aging.203734 (PMC8714166; doi:10.18632/aging.203734)
Supplement: Supplementary Figures [file aging-13-203734-s001.pdf]

SUPPLEMENTARY FIGURES

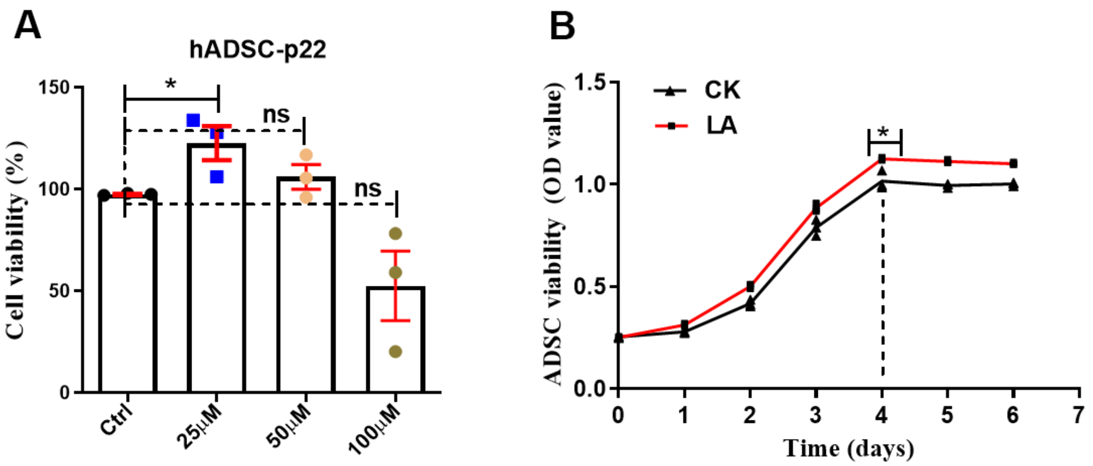

Supplementary Figure 1. Cell viability assay and survival curve analysis to monitor the effect of LA on aged hADSC. (**A**) Cell viability assay. (**B**) Survival curve. The data are presented as mean ± SD of three independent experiments. * p < 0.05, ** p < 0.01 compared to untreated cells.

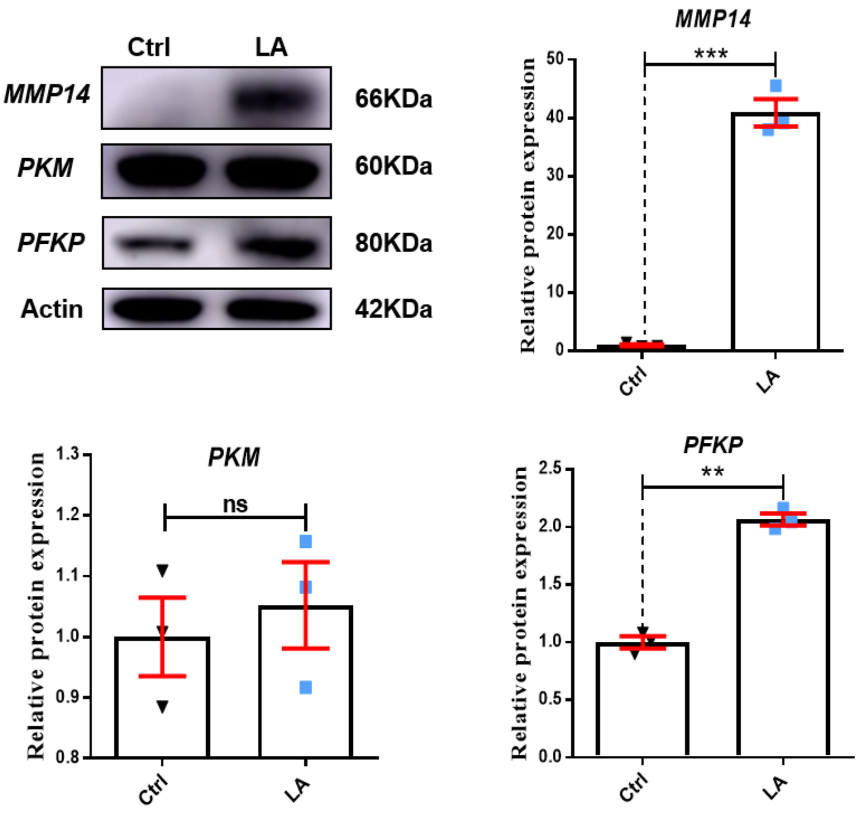

Supplementary Figure 2. Western blot analysis of glycolysis/gluconeogenesis signaling pathway related proteins in hADSCs treated as indicated. Data are presented as the mean ± SD of three independent experiments. *p < 0.05, **p < 0.01, ***p < 0.001 compared with untreated cells.

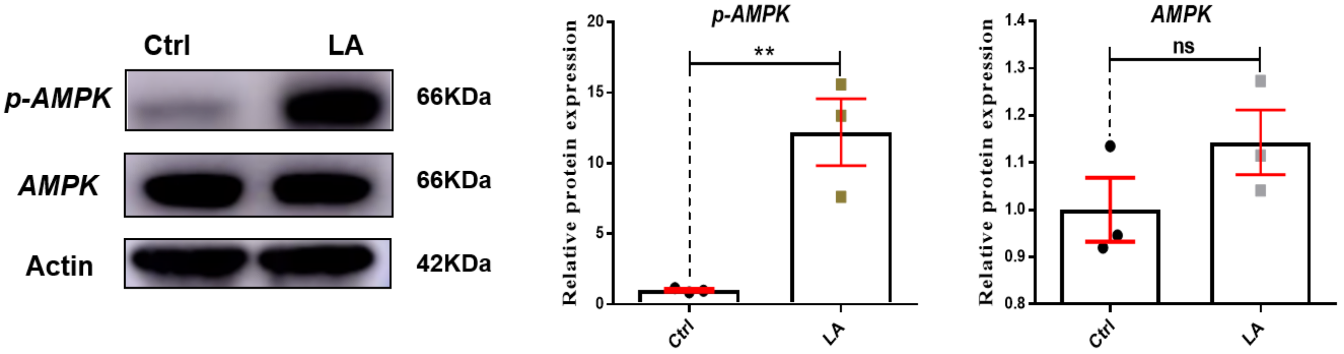

Supplementary Figure 3. Western blot analysis of AMPK protein in hADSCs treated as indicated (LA 24 h treatment). Data are presented as the mean ± SD of three independent experiments. *p < 0.05, **p < 0.01, ***p < 0.001 compared with untreated cells.

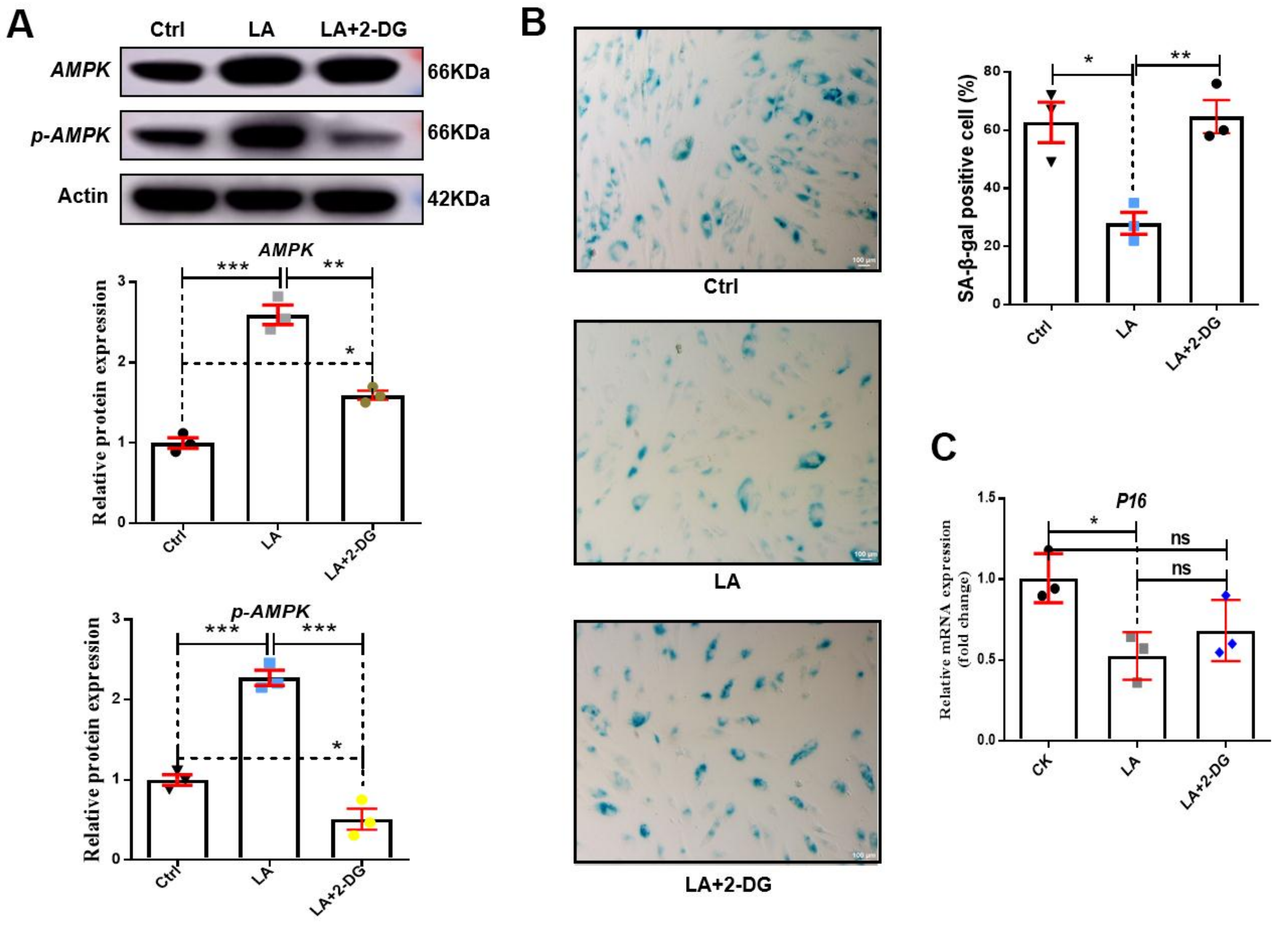

Supplementary Figure 4. Influences of LA on senescence of hADSCs can be reversed by glycolysis inhibitor 2-DG. (**A**) Western blot analysis of *AMPK* protein in hADSCs treated as indicated (LA treatment 80 h). (**B**) SA-β-gal staining of hADSCs (Scale bar: 100 μm) (LA treatment 48 h). The relative intensity of ROS was detected by a microplate reader. (**C**) qRT-PCR of mRNA levels of p16 gene (LA treatment 48 h). Data are presented as the mean ± SD of three independent experiments. *p < 0.05, **p < 0.01, ***p < 0.001 compared with control cells.
